# A Hyaluronic Acid Functionalized Self-Nano-Emulsifying Drug Delivery System (SNEDDS) for Enhancement in Ciprofloxacin Targeted Delivery against Intracellular Infection

**DOI:** 10.3390/nano11051086

**Published:** 2021-04-22

**Authors:** Rabia Arshad, Tanveer A. Tabish, Maria Hassan Kiani, Ibrahim M. Ibrahim, Gul Shahnaz, Abbas Rahdar, Misook Kang, Sadanand Pandey

**Affiliations:** 1 Department of Pharmacy, Quaid-i-Azam University, Islamabad 45320, Pakistan; rabia.arshad@bs.qau.edu.pk (R.A.); marria.h.kyani@gmail.com (M.H.K.); 2 UCL Cancer Institute, University College London, London WC1E6DD, UK; t.tabish@ucl.ac.uk; 3 Department of Pharmacology, College of Medicine, King Abdulaziz University, Jeddah 21589, Saudi Arabia; imibrahim1@kau.edu.sa; 4 Department of Physics, Faculty of Science, University of Zabol, Zabol 538-98615, Iran; 5 Department of Chemistry, College of Natural Science, Yeungnam University, 280 Daehak-Ro, Gyeongsan 38541, Gyeongbuk, Korea

**Keywords:** biofilms formation, pharmacokinetics, drug delivery, anti-bacterial activity

## Abstract

Ciprofloxacin (CIP), a potent anti-bacterial agent of the fluroquinolone family, shows poor solubility and permeability, thus leading to the development of intracellular pathogens induced multi-drug resistance and biofilms formation. To synergistically improve the biopharmaceutical parameters of CIP, a hyaluronic acid (FDA approved biocompatible polymer) functionalized self-nano emulsifying drug delivery system (HA-CIP-SNEDDS) was designed in the present study. SNEDDS formulations were tested via solubility, droplet size, zeta potential, a polydispersity index, thermodynamic stability, surface morphology, solid-state characterization, drug loading/release, cellular uptake, and biocompatibility. The final (HA-CIP-SNEDDS) formulation exhibited a mean droplet size of 50 nm with the 0.3 poly dispersity index and negative zeta potential (−11.4 mV). HA-based SNEDDS containing CIP showed an improved ability to permeate goat intestinal mucus. After 4 h, CIP-SNEDDS showed a 2-fold and HA-CIP-SNEDDS showed a 4-fold permeation enhancement as compared to the free CIP. Moreover, 80% drug release of HA-CIP-SNEDDS was demonstrated to be superior and sustained for 72 h in comparison to free CIP. However, anti-biofilm activity of HA-CIP-SNEDDS against *Salmonella typhi* was higher than CIP-SNEDDS and free CIP. HA-CIP-SNEDDS exhibited increased biocompatibility and improved oral pharmacokinetics as compared to free CIP. Taken together, HA-CIP-SNEDDS formulation seems to be a promising agent against *Salmonella typhi* with a strong targeting potential.

## 1. Introduction

*Salmonella typhi* is a zoonotic intracellular pathogen and its invasion into eukaryotic cells leads to the formation of intracellular systemic infectious typhoid fever, which is a major public health problem worldwide [1,2,3]. *Salmonella typhi* soon after intracellular internalization destroys microfold cells (M cells) of the intestine to cross biological barriers via entering macrophages and mesenteric lymph nodes, followed by the dissemination of infected macrophages into the liver, spleen, and bone marrow, which in turn establishes chronic infections [4,5]. *Salmonella typhi* while residing in the macrophages matures a shielding membrane termed as *Salmonella* containing vacuole (SCV) for its replication, nourishment, and ultimate survival [3,6]. *Salmonella typhi* infected macrophages are prone to shed this notorious bacterium in liver and gall bladder, thereby developing the resistant biofilms [7,8]. Antimicrobial agents, therefore, show poor permeability across the host cell membrane before reaching the targeted infection reservoirs [9]. Ciprofloxacin (CIP) has an excellent sterilizing effect on the biliary and fecal reservoirs of chronic carriers of *Salmonella typhi* in comparison to conventional antibiotics such as ampicillin and penicillin [10]. However, existing oral formulations of CIP have limited success in crossing biological and cellular barriers because of low solubility, poor permeation, and non-specific distribution [11]. As CIP has been characterized as biopharmaceutical classification system (BCS class IV) drug (low solubility/low permeability), the development of oral formulations capable of crossing multi-barriers with high penetration, specific distribution and targeted/selective delivery against *Salmonella typhi* infection remains challenging [12,13]. Driven by these needs, in the present study we develop mucopermeating and HA functionalized SNEDDS for the sustained release as well as for improved solubility and oral bioavailability of CIP [14]. SNEDDS has recently emerged as an effective and suitable alternative for the controlled and targeted delivery of a wide range of drugs [15,16]. SNEDDS are isotropic mixtures of oils, surfactants, and cosurfactants that form a fine oil in water nano emulsion upon mild agitation in GI media [17]. SNEDDS orient nano emulsions in the range of 20 and 200 nm upon dilution. In comparison to other nanocarrier system such as liposomes, noisome, and transferosomes, the synthesis of SNEDDS is facile, straightforward, and cost-effective. Hyaluronic acid (HA) is a Food and Drug Administration (FDA) approved marine-derived polymer of notable medicinal importance [18]. HA is made up of the repeating disaccharide units of N-acetylglucosamine and glucuronic acid. Moreover, HA is a constituent of the extracellular matrix, and binds specifically to CD44 and Toll-like receptor 4 (TLR4) [19]. In intestinal inflammatory infections such as *Salmonella typhi,* the overexpression of TLR4 and CD44 receptors has evidently been reported [20]. Hence, HA is a good choice when used at correct ratios and doses in achieving targeted anti-bacterial actions via enhancing phagocytic activity and promoting the entry of CIP into host intestinal cells [21]. In the present study, for the first time, chemical attachment of CIP into HA (via carbodiimide chemistry) to further develop HA-CIP-SNEDDS formulation has been developed in order to facilitate the muco-permeation, anti-bacterial action, and efficacious targeted delivery against *Salmonella typhi* pathogenesis.

## 2. Materials and Methods

### 2.1. Materials

Ciprofloxacin base (molecular weight: 331.34 Da), hyaluronic acid (LMW HA, MW 500 kDa), N-hydroxy succinimide (NHS), 1-Ethyl-3-(3-dimethylaminopropyl)carbodiimide hydrochloride (EDAC),cremophore RH 40, tween 80, PEG 400, PEG 200, artificial mucin, oleic oil, linseed oil, caproyl 90, labrafil M, mineral oil, dulbecco’s modified eagle’s medium (DMEM), rhodamine, crystal violet, congo red, 4′,6-diamidino-2-phenylindole(DAPI), and fluorescein isothiocyanate (FITC) dye were purchased from Sigma Aldrich, Germany. Cystamine dihydrochloride was purchased from Avonchem, New Hampshire, UK) United Kingdom. All solvents used were of analytical grade.

### 2.2. Synthesis of Hyaluronic Acid (HA)-Ciprofloxacin (HA-CIP) Conjugate

Pure CIP was initially successfully linked to HA via amide bond formation via protection of acidic group of CIP by methylation in 24 h reflux reaction, followed by the addition of HA through EDAC/NHS coupling. Pure carboxylic acid protected CIP was added in the preformed reaction mixture, and 0.1% polymeric solution of HA was prepared, and its pH was adjusted to 5 by adding 5M NaOH [22]. Furthermore, EDAC (500 mg) was added, followed by continuous stirring for 2 h to activate carboxyl groups of HA. After stirring the equimolar solution, 24 h of stirring occurred at 10 °C. The final filtered drug-polymer conjugate was then dialyzed (molecular weight cut off: 14,000–26,000) three times using double distilled water. The dialysis has to be done against high salt (1 M NaCl) followed by lyophilization (Christ Alpha 1-2 LD plus, Gernsheim, Germany). The obtained solution was lyophilized and the product obtained was characterized using Size exclusion chromatography and the amount of CIP conjugation was determined using HPLC and ^1^H NMR spectra of HA-CIP conjugate, using DMSO solvent.

#### 2.2.1. Drug Solubility Studies in Oil/Surfactant/Co-Surfactant Mixtures

The Shake flask method was utilized to determine the solubility of CIP in different oils (labrafil M, capryol 90, oleic oil), surfactants (Cremophore RH40, Tween 80, Span 20), and co-surfactants (PEG 400, PEG 200, PEG 600, Span 80) from Sigma-Aldrich (St. Louis, MO, USA). In a 10 mL homogenous mixture of SNEDDS excipients, an excess concentration of CIP was added and vortexed for 5 min. Vortexed mixture was shaken in the shaking water bath at a rate of (50 strokes/minute) at 37 °C for 24 h for aided drug solubilization. The centrifugation of the resulting homogenous mixture was done at 2800 g for 15 min and supernatant was separated. Dilution of the collected supernatant was performed in a mixed solvent system of ethanol and water (1:1, *v*/*v*). Afterwards, filtration was done through a Millipore filter membrane with a 0.45 µ pore size. CIP in each of the oil/surfactant/co-surfactant mixtures was quantified at the absorbance of 276 nm via UV-visible spectrophotometer (Beckman Coulter, Brea, CA, USA) [23].

#### 2.2.2. Preliminary Selection of Excipients

A solubility study of CIP was evaluated in numerous oils, surfactants, and co-surfactants. The solubilization compatibility results of CIP are shown in Figure S1B,C. Pure CIP showed the highest solubility in capryol 90 (471.35 ± 0.97 mg/mL) as compared to other oils, as shown in Figure S1B. Therefore, capryol 90 was selected as an oil phase for formulation of SNEDDS. CIP was less soluble in Cremophore RH40 than in tween 80 (15.58 ± 2.98 mg/mL). It was evident that oleic oil was emulsified with tween 80 in a shorter period than cremophore RH40. Following the results, tween 80 was selected as a surfactant for designing the formulation and PEG 200 was evaluated for co-surfactant selection. However, it was shown that CIP was ideally soluble in oleic oil. Tween 80 as a surfactant and PEG as a co-surfactant are shown in Figure S1C. It was evident from the results that the combination of oleic oil with tween 80 and PEG 200 leads to a low number of flask inversions and maximum % age of transmittance, thus making an ideal combination of SNEDDS formulation.

#### 2.2.3. Screening of Surfactant/Co-Surfactant Emulsification Capability

Different surfactants as well as co-surfactants were analyzed on the principle of % transmittance and efficient emulsion capability. Surfactant (200 mg) and co-surfactant (100 mg) were mixed in 300 mg of oil phase in a 1:1 ratio at 40 °C for homogenization of the isotropic mixture. The homogenized isotropic mixture was diluted in 10 mL distilled water. The emulsifying capability of the emulsion was checked through a number of inversions of the flask.

#### 2.2.4. Pseudo Ternary Phase Diagram

Pseudo-ternary phase diagram was plotted to distinguish and optimize the self-nano emulsification region of oil, surfactant, as well as cosurfactant. Moreover, each optimized concentration of oil, surfactant, and cosurfactant represented a triangle apex. Pseudo-ternary plots are based on different ratios of the surfactant (30–40%), co-surfactant (20–30%), and oil (10–50%) to make a cumulative 100% outcome using MODDE software (version 12.1). The ideal SNEDDS formulation was determined by fluctuating the concentrations of excipients and sustaining the polymer concentration. A SNEDDS mixture (100 mg) was dissolved in 10 mL distilled water at 37 °C to develop stable nano emulsion. As prepared nano emulsion was evaluated in terms of droplet size, polydispersity index, and percentage of transmittance [24].

#### 2.2.5. Development of CIP Loaded Polymeric SNEDDS

Optimum conditions for harvesting ideal SNEDDS include high percentage transmittance, emulsification capability, size less than 200 nm, and a polydispersity index less than 0.5. Therefore, an appropriate ratio of HA polymer, oil, surfactant, and co-surfactant was mixed followed by probe sonication (20K HZ) for 5 min at 50 °C. After complete mixing of all above-mentioned excipients, the excess quantity of CIP was added, and the reaction mixture was vortexed for aided solubilization. Furthermore, the resulting polymeric HA-CIP-SNEDDS was cooled and stored at the room temperature to observe its stability [25].

### 2.3. Physicochemical Characterization of HA-CIP-SNEDDS

The developed HA-CIP-SNEDDS were physicochemically characterized in terms of transmittance, dispersibility, solubility, robustness of dilution, and cloud point. Detailed information of the following methods is provided in the supplementary materials.

#### 2.3.1. Size, Polydispersity Index and Zeta Potential

SNEDDS dilutions in the distilled water in the ratio of 1:100 were followed by sonication with a probe sonicator for 1 min to form nano emulsion. Furthermore, formed nano emulsions were examined for the size, polydispersity index, and zeta potential through a zeta sizer [26].

#### 2.3.2. Surface Morphology via Transmission Electron Microscopy (TEM) Analysis and Scanning Electron Microscopy (SEM)

A droplet of SNEDDS nano emulsion formulation was placed on the carbon-coated copper stubs and stained with phosphotungstic acid solution (2%*w*/*w*) operational under vacuum at 40 mA for 15 sec [25]. SEM and TEM of the HA-CIP SNEDDS and HA-SNEDDS was carried out using (FEI Nova Nano SEM 450, St. Louis, MO, USA) SEM equipped with TEM by employing the samples on the carbon-coated copper grid smudged by a drop of 1% ammonium molybdate solution.

#### 2.3.3. Thermodynamic Stability Tests

The thermodynamic stability of the SNEDDS was determined by subjecting it to various stress conditions including centrifugation of optimized diluted SNEDDS in the ratio of (1:100) at 20,142 g for 45 min in a centrifuge machine. Centrifugation was followed by incubation of nano formulation at 4 °C and 45 °C (heating-cooling cycles) and incubating of samples between −20 °C and 25 °C (freeze-thaw cycles) for 48 h to observe any evident changes in clarity, drug precipitation, and phase separation with the naked eye [27].

#### 2.3.4. FTIR, DSC and XRD Analysis

FTIR analysis of pure CIP, HA-CIP SNEDDS, and HA-SNEDDS was performed by using an FTIR spectrophotometer ((Bruker α, St. Louis, MO, USA) to evaluate the electrostatic interactions between different combinations. A suitable concentration of samples in powdered form was mixed with potassium bromide (KBr) to form a pallet by smearing 200 psi mechanical pressure. Pellets were then scanned in the wavenumber of 500–4500 cm^−1^. Samples were analyzed for qualitative properties and thermal behavior over a wide range of temperature series of 50–300 °C at a quantified rate of 10 °C/min under atmospheric nitrogen (50 KPa) pressure. XRD was performed for CIP, HA-CIP SNEDDS, and HA-SNEDDS by adjusting the Ɵ between of 20–70° with a step size of 0.5505 and ƛ (Cua-k).

### 2.4. In Vitro Dissolution

An HA-CIP-SNEDDS dissolution mechanism was determined through simple diffusion for 72 h at pH 5.0 to mimic the phagosome pH because CIP has antibacterial activity against intestinal infections and notorious bacteria involved in serious intestinal infection i.e., resistant *Salmonella typhi* hijack host endosome to proliferate. HA-CIP-SNEDDS were immersed in 200 mL of PBS (pH 5) in the beaker followed by shaking at 37 °C and 150 rpm. However, 3 droplets of 15 mM glutathione were injected in the PBS (pH 5) to maintain the sink conditions because of the very low solubility of pure CIP. Furthermore, a sample of 3 mL SNEDDS formulations was withdrawn from dissolution media and centrifuged at 14,000 rpm for 30 min. The supernatant separated from the centrifugation was used for CIP quantification and the pellet was again suspended in the same amount of fresh media (3 mL) equilibrated at 37 ± 0.5 °C and poured back to the respective dissolution media. The presence of CIP in the supernatant was quantified by UV-Visible double beam spectrophotometer at 276 nm [28].

### 2.5. Ex Vivo Permeation

Permeation was evaluated using the everted sac method on the intestine of goat. The intestine, after being washed with Krebs’s ringer solution, was then cut into 5 cm small segments. For the everted sac, a glass rod was passed through one open end of the intestinal segment and then the segment was gently rolled down over it. One side of the sac was tied and loaded with drug suspension (control), CIP-SNEDDS, HA-SNEDDS, and HA-CIP-SNEDDS with syringe needles and the remaining end was also tied in the same manner. The SNEDDS incorporated intestinal segments were then engrossed in the beaker containing 10 mL of the Krebs’s ringer solution at 37 °C. Samples were pipetted out and an equal volume of Krebs’s solution was replaced at specified time intervals. The samples were then collected, evaluated, and quantified using UV spectrophotometer [29]. Apparent permeability (Papp) can be calculated using the following formula:

*Apparent Permeability* (μg/cm^2^) = Q/Act. (1)

By assuming intestine characteristics, a cylinder, MSA was calculated using the formula:

*Mucosal Surface Area* (cm^2^) = *Circumference* (*π* × *diameter*) × *Length*. (2)

### 2.6. In Vitro Mucoadhesion Studies

Rheology of the SNEDDS was determined using cone-plate viscometer with the power of 150 VA and Volt/Freq: 90–260V–50/60HZ). Nano emulsion (1 mL) was further added into 5 mL of mucin solution. Furthermore, the incubation of reaction mixture was done at 37 ± 0.5 °C and the viscosity of the final mixture was observed at regular intervals of 1, 2, and 4 h at shear rate of 60 s^−1^.

Δη = η_mix_ − η_muc_ (3)

η_mix_ = Viscosity of mucin-nano emulsion mixture.
η_muc_ = Viscosity of mucin dispersion.

### 2.7. Drug Entrapment Efficiency

HA-CIP-SNEDDS and HA-SNEDDS (2 mL) based nano emulsions entrapment efficiency was determined. Content of free CIP in nano emulsion was separated via the ultrafiltration method (3500 D) and centrifugation at 20,412 g at 4 °C for 1 h. The drug contents in the supernatants were calculated by determining the absorbance at 276 nm through a UV-Visible (UV-vis) double beam spectrophotometer [30]. The drug entrapment efficiency was calculated via using the following equation:

Entrapment efficiency (%) = *Wt*/*Wx* × 100 (4)

where *Wt* = Total amount of CIP within the prepared SNEDDS; *Wx* = Total amount of CIP added for the SNEDDS preparation.

### 2.8. In Vitro Hemolytic Assay via Spectrophotometry

Hemolysis assay was performed to determine the hemocompatibility of HA-CIP-SNEDDS and HA-SNEDDS. Fresh human blood 5 mL from healthy volunteers (with the permission of volunteers) was washed three times with normal saline solution (0.9% NaCl), and diluted with Dulbecco phosphate buffer saline (PBS) in the mixing ratio of 1:9. Different concentrations of SNEDDS formulations (1–3 mg/mL) were added into the diluted blood and incubated at 37 °C. After 24 h of incubation, samples were centrifuged at 1008 for 10 min at 4 °C and supernatants were collected [31]. Furthermore, the absorbance of the supernatants was evaluated via reading micro titer plate reader at 540 nm. The hemolysis rate can be determined by the following equation:

(5) $$\%\;\mathrm{hemolysis}=\frac{\mathrm{absorbance}\;\mathrm{of}\;\mathrm{sample}-\mathrm{absorbance}\;\mathrm{of}\;\mathrm{negative}\;\mathrm{control}}{\mathrm{absorbance}\;\mathrm{of}\;\mathrm{positive}\;\mathrm{control}-\mathrm{absorbance}\;\mathrm{of}\;\mathrm{negative}\;\mathrm{control}}\;\times \;100$$

### 2.9. Biofilm Dispersion Assay

Biofilm assay was performed by growing *Salmonella typhi* resistant strains (obtained from NIH, Islamabad, Pakistan) in LB broth at 37 °C in polystyrene 12 well microtiter plates and incubation was done for 2 days. After 2 days, SNEDDS were added in the wells of microtiter plates and were again subjected to 2 days incubation at 37 °C. Un-treated biofilms was used as a control. After the incubation period of 2 days, the wells were washed thrice with PBS to remove extracellular bacteria, and overnight inversion of the plates was done at 37 °C for drying purposes. After drying, staining of the *Salmonella typhi* biofilms with 2% crystal violet was completed for 2 min at the laboratory temperature of 37 °C. The plates were rinsed with double distilled water, dried, and captured. Isopropyl alcohol 70% was used to extract the amount of crystal violet bounded to *Salmonella typhi* biofilms by measuring the absorbance at 595 nm [32].

### 2.10. Cellular Uptake Study

The uptake of SNEDDS by seeded macrophages RAW 264.7 cells was estimated using fluorescent microscopy. Macrophages RAW 264.7 cells at a density of 5000 cells/well were replenished with DMEM media, 10% (*v*/*v*) FBS and 1% antibiotics and incubated in CO_2_ incubator at 37 °C to attain the confluency of 70–80%, followed by the replacement with fresh media. Moreover, FITC labeled SNEDDS were added into the 96 well plates to be internalized by the cells under 5% CO_2_ at 37 °C for 4 h. After internalization of FITC labeled SNEDDS, cells were rinsed with PBS for removing uninternalized nanoparticles. Fluorescent imaging of the cells was achieved using a constant exposure time with a fluorescent microscope [33].

### 2.11. In Vitro Cytotoxicity

MTT assay was used to assess the in vitro cytotoxicity and biocompatibility of CIP-SNEDDS, HA-CIP SNEDDS and blank SNEDDS. Macrophages RAW 264.7 were seeded at a density of 5000 cells/well in polystyrene 96 well plates and DMEM media. Media was accompanied with 10% (*v*/*v*) FBS and 1% antibiotics were supplemented and the cells were incubated in a CO_2_ incubator at 37 °C for 4 h. SNEDDS and 10 mg CIP was diluted and resuspended in DMEM media in the concentration ranges of 10, 20, 30, 40, 50, and 100 µg/mL for 24 h. Blank cells in 96 well plates were without MTT and the positive control was accompanied with triton X-100. Furthermore, incubation of all the culture plates inoculated with SNEDDS formulations was done in the CO_2_ incubator at 37 ± 0.5 °C for 24 h, followed by replenishment with fresh media, 500 µg/mL MTT/PBS solution, and incubation for 4 h. Interaction of MTT and viable cells resulted in the formation of violet colored formazan crystals. Therefore, to dissolve formazan crystals, DMSO (100 µL) was added to each well and absorbance was recorded at 540 nm via a multiplate reader [34]. (Perkin Elmer, Waltham, MA, USA). The percentage of cell viability was calculated using the following equation:

(6) $$\%\;\mathrm{cell}\;\mathrm{viability}=\frac{\mathrm{Absorbance}\;\left(\mathrm{treated}\;\mathrm{cells}\right)-\mathrm{Absorbance}\;\left(\mathrm{blank}\right)}{\mathrm{Absorbance}\;\left(\mathrm{non}-\mathrm{treated}\;\mathrm{cells}\right)-\mathrm{Absorbance}\;\left(\mathrm{blank}\right)}.$$

### 2.12. Antibacterial Activity

The antibacterial activities of control, CIP, HA, and HA-CIP-SNEDDS were evaluated by incubating formulations with bacterial strain cells PBS (pH 7.4). Next, 3 × 10^4^ cells ml^−1^ density of *Salmonella typhi* strains was mixed with SNEDDS and incubated at 37 °C for 3 h. After incubation, solidified LB agar plat was impregnated with 100 μL of the bacterial suspension. The colony-forming units (CFU) were counted after 18 h of incubation [35]. The sterilization rate can be calculated by the following formula:

Sterilization rate % = (C_0_ − C)/C_0_ × 100 (7)

where C is the CFU of the experimental group treated with CIP and polymeric SNEDDS and C0 is the CFU of the control group (Triton X-100) receiving no treatment [36].

### 2.13. In-Vivo Survival Assay against Salmonella Typhi

In-vivo survival assay was conducted on female BALB/c mice (6–8 weeks) divided into 3 groups (n = 5) under the approval of Bioethical Committee Quaid-i-Azam University, Islamabad, Pakistan, following NIH guidelines. Mice were infected with oral *Salmonella typhi* dose of (10^8^ CFU), and symptoms appeared (lethargy, hair loss, raised temperature, and inactivity). After the appearance of symptoms, treatment with control, CIP, and oral SNEDDS (10 mg/kg) was initiated 5 days post-infection to determine the effectiveness of novel SNEDDS treatment in mice accompanying advanced-stage infection [1,36].

### 2.14. In Vivo Pharmacokinetics

In-vivo pharmacokinetics of HA-CIP SNEDDS was evaluated in mice via high-performance liquid chromatography (HPLC) method development. The animal study was conducted in accordance with the Bioethical Committee of Quaid-i-Azam University Islamabad, Pakistan, under assigned protocol of BEC-FBS-QAU2020-267. Healthy mice weighing 30 g were kept in the primate facility with free access to food and water a day before the commencement of the experiment. The mice were divided into two groups (n = 5). Group one was subjected to pure CIP and group 2 was administered with HA-CIP-SNEDDS via an oral gavage needle. Blood samples were withdrawn via a sterile syringe from marginal ear veins of the mice at different time intervals (0.5, 1, 2, 4, 6, 8, 12, and 24 h). The withdrawn samples were then transferred to 1.5 mL Eppendorf tubes containing 100 mL anticoagulant, followed by centrifugation of the blood samples at 4000 rpm for 15 min for separating plasma. Blood plasma was stored at −20 °C until further use. CIP was extracted from the plasma and quantified via the validated HPLC method [37].

### 2.15. Statistical Analysis

Results were analyzed statistically with one-way analysis of variance (ANOVA) by taking a significance value of less than 0.05. All the experiments were performed in duplicates as mean ± SD.

## 3. Results and Discussions

The strategy behind synthesis of novel HA biofunctionalized SNEDDS of CIP via carbodiimide chemistry is to enhance the advanced and highly specified targeted drug delivery against *Salmonella typhi*. As HA is the carrier for macrophage activation, this results in binding and endocytosis.

Moreover, HA resulted in increased synergistic uptake of HA in intestinal inflamed cells. Enhanced recognition by receptor scavenging cells as well as intracellular trafficking facilitated the internalization of HA functionalized SNEDDS of CIP into intestinal epithelial cells, resulting in proficient targeting and eradication of *Salmonella typhi* [38]. Therefore, precise, and innovative selection of *anti-salmonella* drugs and biocompatible polymeric ligands will circumvent oral biological and cellular barriers against *Salmonella typhi.*

### 3.1. Synthesis and Characterization of HA-CIP Conjugate

**Figure 1:**
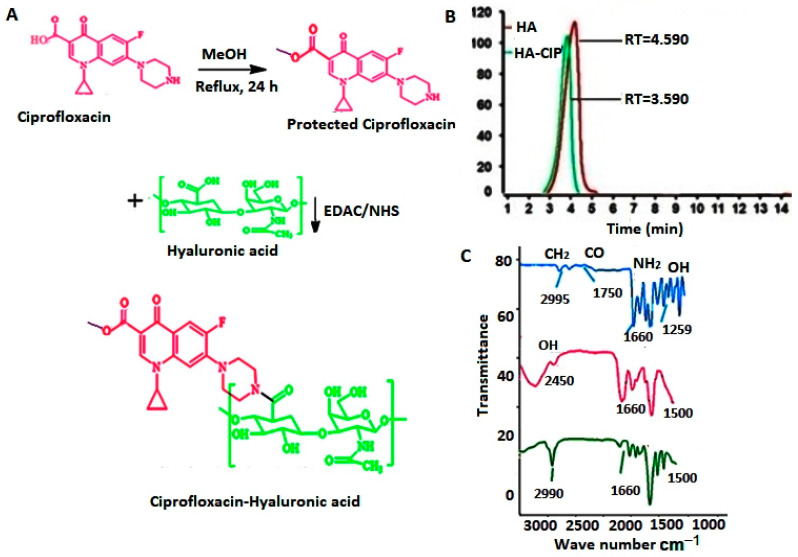
(**A**) Hyaluronic acid conjugated with ciprofloxacin to form Hyaluronic acid-Ciprofloxacin (HA-CIP) conjugate, (**B**) Size exclusion chromatography of HA and HA-CIP conjugate. The decrease in the retention time (RT) of the conjugate peak as compared to HA confirmed the synthesis of the conjugate. (**C**) FTIR spectra of CIP (**a**), HA (**b**) and HA-CIP (**c**).

HA and CIP were conjugated by following the protection of acidic group of CIP for removing amphoteric ions and aided solubility as well as simplest and reproducible carbodiimide chemistry using EDAC/NHS via amide bond formation between the carboxylic group of polymer HA and amino group of CIP [39], as shown in Figure 1A. The successful conjugation of the HA and CIP was confirmed via size exclusion chromatography (Figure 1B) and FTIR (Figure 1C). The decrease in the retention time for HA-CIP conjugate as compared to HA (Figure 1B) demonstrated the synthesis of conjugates, as was also confirmed in previous studies.

**Figure 2:**
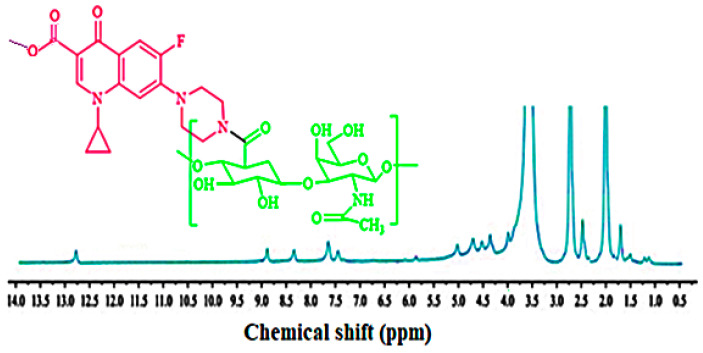
^1^H NMR spectra of HA-CIP conjugate in DMSO.

FTIR spectra of CIP, HA, and HA-CIP has been given in Figure 1C. There was a typical OH stretching peak at 1250 cm^−^^1^, which shows the presence of CIP due to intermolecular hydrogen bonding. CH bond stretching was observed at 2995 cm^−1^ due to aromatic bond stretching. However, bond stretching of CO and NH_2_ in CIP FT-IR spectra was observed at 2995, 1750, and 1650 cm^−1^, respectively. In the case of FTIR spectra of HA, sharp carbonyl stretching at 1600 cm^−1^ was observed. Modification of the polymers with drugs and other functional group entities results in changes in the backbone of the polymer. As far as size exclusion chromatography is concerned, the decrease in the retention time for HA-CIP conjugate as compared to HA demonstrated the synthesis of conjugate, which was also confirmed by previous studies. In NMR, as shown in Figure 2, after the condensation reaction of HA-CIP conjugate, the chemical shifts of NH and aromatic hydrogens of compound HA are in accordance with the signals of compound HA-CIP. The Acidic H peak between *δ* = 12 to 13 ppm indicates the COOH group of the conjugate. However, the sharp peak of NH of piperazine in CIP appeared as a sharp peak near *δ* = 2 ppm. Moreover, the signal of methyl group of the amide moiety appears at *δ* = 2.24 ppm.

### 3.2. Construction of a Pseudo Ternary Phase Diagram

**Figure 3:**
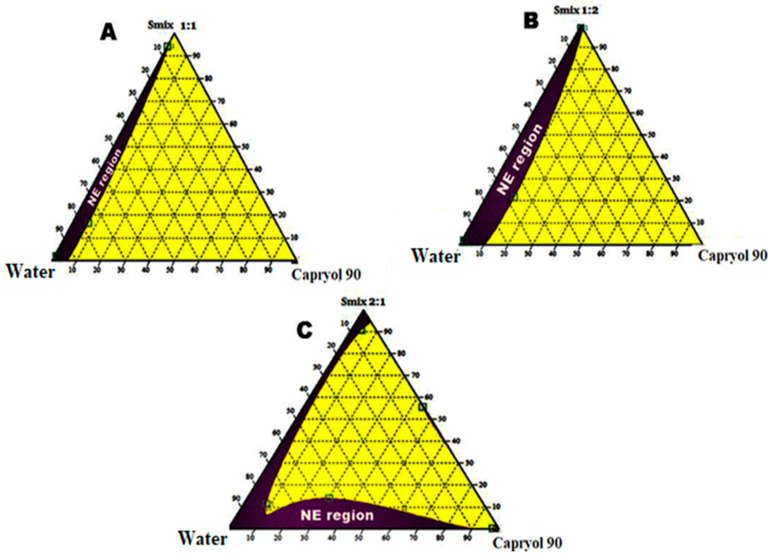
Ternary phase diagram representing the emulsification region. (**A**) With Smix ratio = 1:1, (**B**) Smix ratio = 1:2, & (**C**) Smix ratio = 2:1. purple colored area indicates nanoemulsion and yellow colored area indicates microemulsion.

A ternary phase diagram was developed to identify the self-nano emulsifying region and the optimum concentration and combination of oil, surfactant, and co-surfactant. Ternary mixtures were prepared using excipients having a supreme solubilizing capacity for CIP. Percentage transmittance was selected to illustrate the ternary mixtures as having a good or bad emulsion. By changing concentrations of oil, surfactant, and co-surfactant, various formulations were developed and assessed for SNEDDS physicochemical features. The nanoemulsion with a percentage transmittance of ≥ 85% is considered as a good emulsion. Results exhibited the transparent nanoemulsion region with a purple color in the ternary phase diagram [40]. Different concentrations of surfactants as well as co-surfactants were utilized in the development of SNEDDS and depicted the ternary diagrams with varying concentrations of excipients in the ratios of 1:1, 1:2, and 2:1 shown in Figure 3A–C. With varying ratios, the concentration of capryol 90 oil also 10–50%, while the surfactant and co-surfactant needed to be 30–40% and 20–30%, respectively. The purple colored area represented a crystal-clear nano emulsion and yellow colored area indicates microemulsion. Concentrations of oil, surfactant, as well as co-surfactant were adjusted and optimized and are given in Table 1. The nano emulsion region was greater when the Smix ratio of 2:1 was applied, and results are listed in Table 2.

### 3.3. Preliminary Selection of Excipients

Solubility of the CIP was tested with different oils, surfactants, and co-surfactants and finally capryol 90 (oil), tween 80 (surfactant), and PEG 200 (co-surfactant) were chosen for the formulation, as shown in Figure S1B,C.

### 3.4. Fabrication and Physicochemical Characterization of HA-Based SNEDDS for Delivery of CIP

SNEDDS of CIP were synthesized by loading of CIP, and HA in a blank SNEDDS mixture, as shown in Figure S1A. Our primary approach for designing this research study was to identify the effect of control, HA-CIP-SNEDDS, and HA-SNEDDS on the physicochemical properties of SNEDSS. Excipients were selected on the basis of this optimized ratio of the phase diagram region (1:1, 1:2, and 2:1). Concentrations of oil, surfactant, as well as co-surfactant were adjusted and optimized and are given in Table 1. However, it was obvious from results that all optimized SNEDDS formulations showed the highest transmittance. Fine and stable nano emulsions with no signs of phase separation were obtained even after dilutions with deionized water at the ratios of 1:100 and 1:1000. Optimized SNEDDS formulations were further analyzed in in vitro and ex vivo models. The results of % transmittance, saturation solubility, and cloud point of all polymeric SNEDDS are listed in Table 2.

### 3.5. Surface Morphology

The TEM analysis of CIP-SNEDDS and HA-CIP-SNEDDS revealed the uniform distribution and spherical morphology of SNEDDS in Figure S1E. The zeta size results of polymeric SNEDDS have been summarized in Table 3 and Figure S1A. All the SNEDDS formulations have less than a 40 nm size and PDI values of less than 0.3, demonstrating the high stability of nano formulations. The observed DLS results slightly vary from TEM analyzed nanoparticles because of the differences in the principles of both techniques. SEM analysis of HA-CIP SNEDDS and HA-CIP polymer exhibited a fibrous, dense, and porous morphology (as shown in Figure S1F).

### 3.6. DSC and XRD Analysis

**Figure 4:**
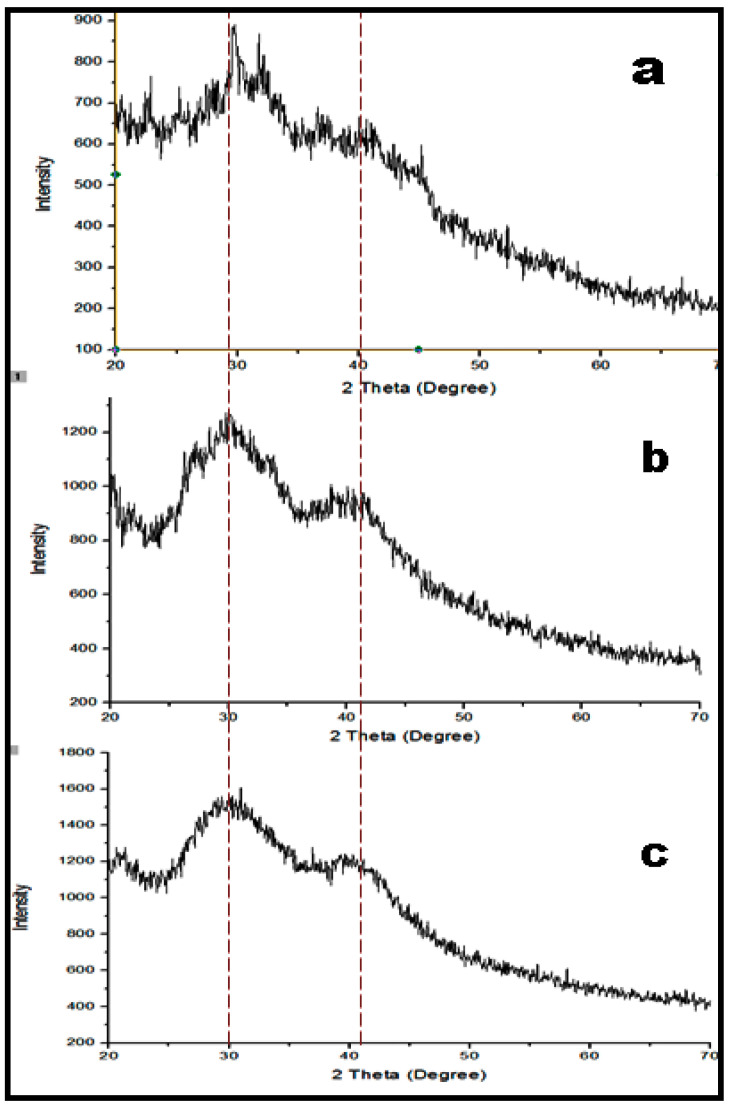
XRD spectra of HA (**a**), CIP (**b**), and HA-CIP (**c**), respectively.

Modification of the polymers with drugs and other functional group entities resulted in the changes in the backbone of polymer. DSC results showed the endothermic peak of CIP at 45 °C while in the case of HA, it showed a characteristic endothermic peak at 25 °C, followed by an exothermic peak at 120 °C. Moreover, a DSC thermogram of HA-CIP showed a characteristic endothermic peak at 26 °C and an exothermic peak at 90 °C. The first endothermic peaks of the drug and polymer denoted the dehydration process and the second endothermic peak showed degradation under atmospheric nitrogen (Figure S2). The XRD pattern in Figure 4 showed the highly crystalline nature of HA polymer as well as the moderate crystalline behavior of the CIP base. However, after their condensation in HA-CIP conjugate, the structure possessed low sharp peaks owing to a reduced concentration of crystalline HA polymer and greater concentration of CIP in the conjugate.

### 3.7. Drug Entrapment Efficiency

**Figure 5:**
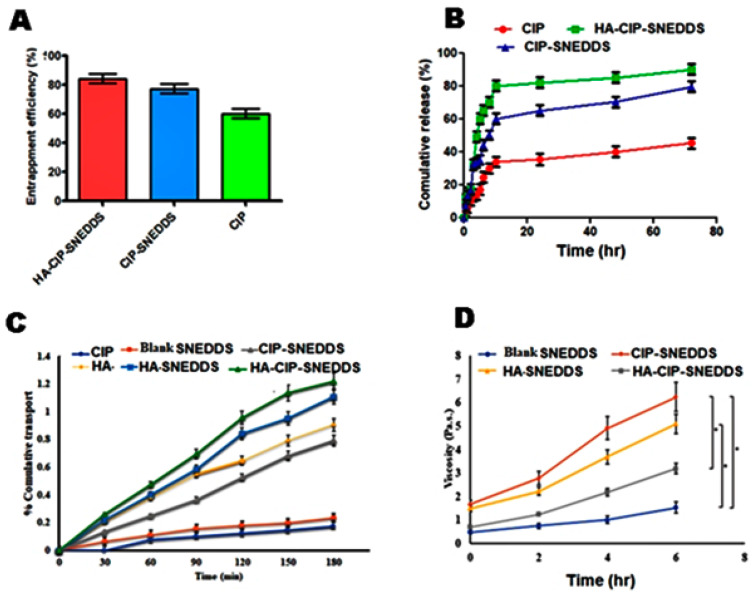
Drug entrapment efficiency of HA functionalized CIP SNEDDS. (**A**) Dissolution studies from CIP and other formulations (**B**). Permeation of CIP and SNEDDS; (**C**) Rheological studies of SNEDDS to determine mucoadhesion and (**D**). Results are listed as duplicates mean ± S.D (*p* < 0.05), and statistically significant differences were evaluated by one-way ANOVA with a significance level of *p* < 0.05.

Drug entrapment efficiency of the polymeric SNEDDS resulted in the improved drug loading of CIP in the polymeric SNEEDS, as shown in Figure 5A. CIP-SNEDDS showed entrapment up to 65% and HA-CIP-SNEDDS showed maximum drug entrapment at 85%. Therefore, it was evident from the results that synthesized HA based SNEDDS proved to be the most promising carriers for encapsulation of Class IV drugs.

### 3.8. In Vitro Dissolution Studies

Dissolution of CIP from polymeric SNEDDS was performed at pH 5.0 to corelate with the endosomal pH in a time dependent manner for 72 h. The drug release pattern of CIP-SNEDDS was burst i.e., about 30% on the first day, while 80% of the drug was released in 72 h. However, HA-CIP_-_SNEDDS followed a sustained release pattern for 72 h, showing 80% release at 72 h, as shown in Figure 5B. The sustained drug release from HA-CIP_-_SNEDDS was due to the steric stabilizing characteristics of HA. CIP-SNEDDS exhibited first order kinetics with an R^2^ value of 0.9672 as shown in Table 4. However, HA-SNEDDS and HA-CIPSNEDDS followed the Korsmeyer–Peppas drug release model. Value of n was < 0.45, and it showed a Fickian diffusion pattern of drug release. The difference observed between the CIP-SNEDDS and HA-CIPSNEDDS release profile was significant (*p* < 0.01).

### 3.9. Ex Vivo Permeation

Permeation of the drug with time is shown in the Figure 5C. The results specified significant improvement in the permeation of CIP in the case of HA-CIP as compared to the CIP solution. HA-CIP-SNEDDS and HA-SNEDDS indicated a 4-fold and 2.5-fold permeation enhancement as compared to the CIP in 3 h, respectively.

### 3.10. In Vitro Mucoadhesion Studies

Results confirmed the 4-fold increase in the viscosity of HA and HA-CIP-SNEDDS as compared to CIP and blank SNEEDS with statistical significance (*p* < 0.001), as shown in Figure 5D. However, the remarkable difference in the viscosity of HA and HA-CIP-SNEDDS was observed owing to the mucoadhesive property of hyaluronic acid, resulting in the interpenetration within polymers. Enhanced mucoadhesion of the HA has also been confirmed through swelling studies.

### 3.11. Salmonella Typhi Biofilm Analysis

**Figure 6:**
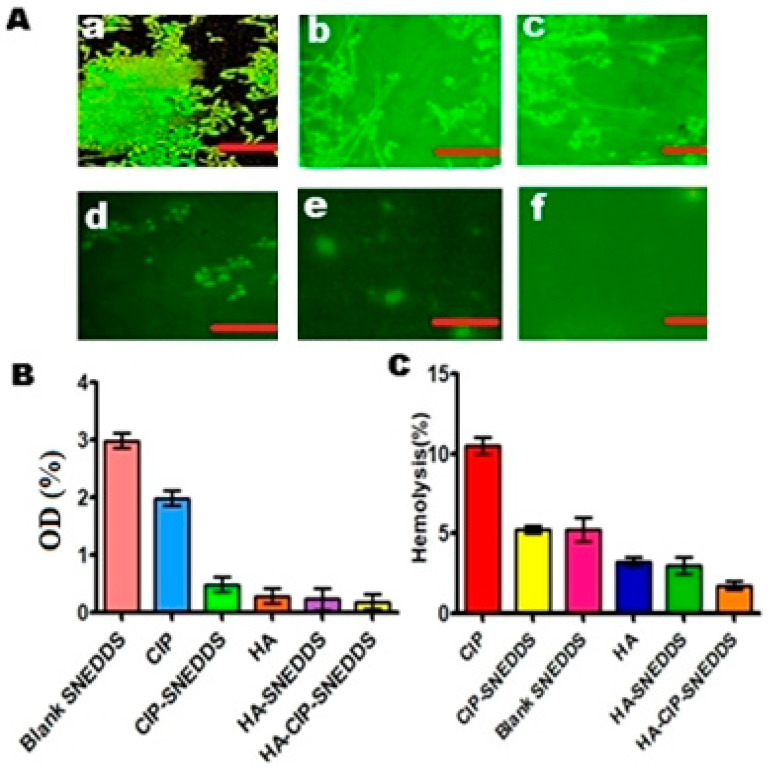
(**A**) Fluorescence imaging of FITC-tagged *Salmonella typhi* biofilms showing the Blank (**a**), CIP (**b**), CIP-SNEDDS (**c**), HA (**d**), HA-SNEDDS (**e**), and HA-CIP-SNEDDS (**f**) growth inhibition of biofilms by treating with SNEDDS (* Scale bars: 50 μm). (**B**) *S. typhi* biofilm dispersion graph of various formulations, (**C**) Determination in vitro hemolysis (%).

*Salmonella typhi* has the ability to form biofilms, which has significantly been encouraged via the prevalence of multi drug resistance and mucosal barriers [28]. In the current study, the results of biofilm dispersion assay illustrated that HA-CIP-SNEDDS were highly proficient in eliminating/dispersing the biofilm co-localization up to the level of 0.02% as compared to the PBS used as control at 1.7% with a statistical difference of *p* < 0.05, as shown in Figure 6A. Fluorescence imaging of FITC tagged-bacterial biofilms also depicted the growth inhibition of *S. typhi* biofilms by HA-CIP-SNEDDS, as shown in Figure 6B. The main reason for the efficient biofilm dispersal using novel HA-CIP SNEDDS was due to targeted anti-bacterial action of HA-CIP SNEDDS.

### 3.12. In Vitro Hemolytic Assay via Spectrophotometry

In vitro hemolysis assay is an essential parameter in evaluating the hemocompatibility of synthesized CIP-SNEDDS. Results demonstrated that HA and HA-CIP-SNEDDS showed 2% and 3% hemolysis as compared to 10% hemolysis of control (Triton X-100) with statistical significance (*p* < 0.001), as shown in Figure 6C. HA and HA-CIP-SNEDDS lie in the safe zone and can be considered as being non-hemolytic. The non-hemolytic nature of HA and HA-CIP-SNEDDS is due to the non-toxic, antioxidant, and anti-virulent properties of HA.

### 3.13. Anti-Bacterial Activity Evaluation

**Figure 7:**
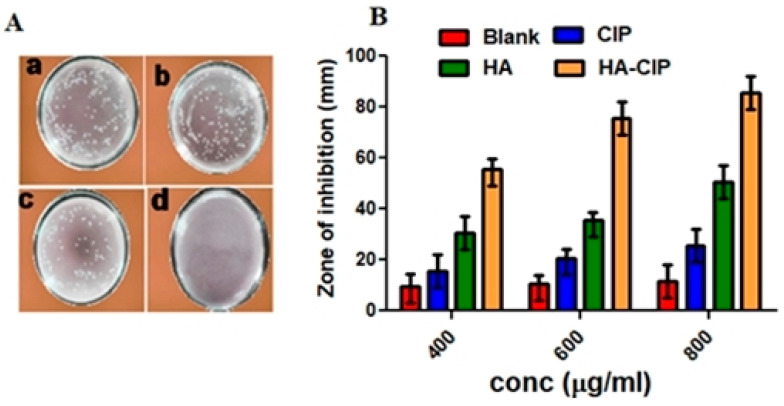
(**A**) Sterilizing effect on *Salmonella typhi* resistant strains via CIP, CIP-SNEDDS, HA-SNEDDS, and HA-CIP SNEDDS (**a**–**d**). (**B**) Zone of inhibition (ZOI) of SNEDDS formulation and CIP.

The antibacterial activities of HA-CIP-SNEDDS against *Salmonella typhi* strains were evaluated by counting the colony. As shown in Figure 7A, the sterilizing rate of CIP was only 9 ± 3.25%. After adding the final polymer, HA-CIP-SNEDDS, the antibacterial activity increased up to 99.55 ± 0.5%. From the results of inhibition/sterilization of *Salmonella typhi*, the zone of inhibition of HA-CIP can be determined. HA-CIP-SNEDDS resulted in maximum bactericidal effects as the concentration of the drug increased (as shown in Figure 7B). The main reason behind this good anti-bacterial activity is due to the targeted killing of *Salmonella typhi* via hyaluronic acid mediated ligand causing synergistic anti-bacterial actions.

### 3.14. Macrophages Uptake by SNEDDS

**Figure 8:**
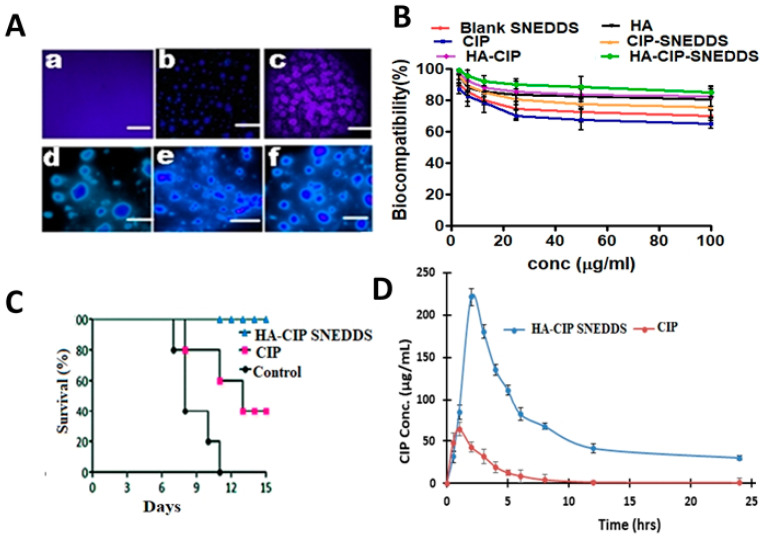
(**A**) FITC tagged step wise increase in florescence from Blank-PBS (**a**), Blank SNEDDS (**b**), CIP-SNEDDS (**c**), HA (**d**), HA-SNEDDS (**e**), and HA-CIP-SNEDDS (**f**) * Scale bars: 50 μm, (**B**) Biocompatibility assay, (**C**) In-vivo survival assay of SNEDDS, (**D**) In vivo pharmacokinetics. Results of all experiments are listed as mean ± S.D.

A macrophage uptake study of nanoparticles was performed to estimate the transport and internalization of SNEDDS formulations into macrophages. Results revealed that FITC-HA-CIP_-_SNEDDS treated macrophages resulted in maximum green fluorescence as compared to the FITC-blank SNEDDS, as shown in Figure 8A. High fluorescence images indicated the proficient and advanced recognition of hyaluronic acid by the scavenger receptors of macrophages in the case of infection [41]. This was because in the case of *S. typhi* infection, *S typhi* fimbriae needed sugar moiety for its replication. Therefore, hyaluronic acid can be of dual importance and provides an efficient ligand to treat and target the intracellular *Salmonella typhi* infection.

### 3.15. In Vitro Cytotoxicity

Cell viability of SNEDDS formulations with modified polymeric system was greater than for CIP-SNEDDS, as shown in Figure 8B. Results obtained after the analysis of in vitro cytotoxicity via MTT assay confirmed the non-cytotoxic nature of HA-CIP SNEDDS as compared to pure CIP. HA-CIP-SNEDDS showed cell viability of 96% at increased drug concentrations as compared to the CIP SNEDDS showing a 65% statistical significance (*p* < 0.05). Therefore, the safety profile of the HA-CIP SNEDDS is owed to the addition of biocompatible, biodegradable, and entero-protective HA [42].

### 3.16. In Vivo Survival Assay against Salmonella Typhi

*Salmonella typhi* can invade intestinal barriers and disseminate into the blood stream via lymphatics into liver and spleen. However, results illustrated that HA-CIP_-_SNEDDS showed no mortality of *Salmonella typhi* infected mice, i.e., all mice survived as shown in Figure 8, owing to targeted delivery [43].

### 3.17. In Vivo Pharmacokinetics

In-vivo pharmacokinetics of HA-CIP and CIP are shown in Figure 8D and pharmacokinetic parameters are given in Table 5. Significant changes were observed in the pharmacokinetic properties of polymeric SNEDDS. HA-CIP-SNEDDS showed a Cmax value of 54.5 μg/mL with T max of 3 h compared to the pure CIP solution C max of 54.30 μg/mL with T max of 1 h. Cmax is the highest concentration of a drug in the blood after the drug dose is administered and Tmax is the maximum time required to reach Cmax. The elimination half-life of HA-CIP-SNEDDS was 16 h against 4 h for CIP, indicating a 4-fold improvement. AUC0-t, AUC0-inf, and AUMC0-inf in the case of HA-CIP-SNEDDS were found to be 1562.83 μg/mL × h, 2124.50 μg/mL × h, and 36,794.5 μg/mL × h^2^, respectively. However, for CIP, the values for the respective parameters were found to be 210.75 μg/mL × h, 225.84 μg/mL × h, and 900.65 μg/mL × h^2^. Increased AUC showed the success of self-nano emulsions in the transportation of drugs into the systemic circulation.

## 4. Conclusions

Hyaluronic acid (HA) based SNEDDSs developed from HA, capryol 90, tween 80, and PEG 200 were investigated as potential carriers for ciprofloxacin enhanced delivery against the intracellular pathogens like *Salmonella typhi*. As-prepared HA-CIP-SNEDDSs resulted in preferential internalization by infected macrophages cells via showing sharp fluorescence. The 80% release of the ciprofloxacin in 72 h from an HA-CIP-SNEDDS established an enhanced *anti-salmonella* activity, thus permitting a reduction in antibiotic dose requirements. Furthermore, the HA-CIP-SNEDDS showed high biocompatibility as compared to the non-polymeric SNEDDS. In summary, the present study indicates that the mucoadhesive SNEDDS seems to be a striking carrier system both for reducing the prospects of drug resistance and to achieve good therapeutic outcomes.

## Figures and Tables

**Table 1:** Different formulation mixtures with varying concentrations of oil, surfactant, and co-surfactant.

Formulations	Surfactant to Co-Surfactant Ratio (Km Ratio)	Capryol 90 (% *w*/*w*)	Tween 80 (% *w*/*w*)	PEG 200 (% *w*/*w*)	CIP (% *w*/*w*)	Polymer (% *w*/*w*)
1	1:1	10	44	44	1	1
2	1:1	20	39	39	1	1
3	1:1	30	34	34	1	1
4	2:1	30	45.3	22.7	1	1
5	2:1	49	26	23	1	1
6	2:1	50	37	11	1	1
7	1:2	20	26	52	1	1
8	1:2	30	22.7	45.3	1	1
9	1:2	40	19.3	38.7	1	1

**Table 2:** Transmittance (%), saturation solubility, and cloud point of polymeric SNEDDS. The results are listed as mean ± S.D (n = 3).

CIP-Loaded Formulations	Transmittance (%)	Saturation Solubility (mg/mL)	Cloud Point (°C)
HA-SNEDDS	89.5 ± 0.05	12.05 ± 0.08	79 ± 0.56
HA-CIP SNEDDS	91.5 ± 0.02	11.01 ± 0.15	78 ± 2.78

**Table 3:** Zeta sizing results of CIP-loaded nano-formulations. The results are listed as mean ± S.D (n = 3).

CIP-Loaded Formulations	Z-Average (d. nm)	PDI	Zeta Potential (mV)
HA-SNEDDS	50 ± 0.567 nm	0.3 ± 0.004	−8.60 ± 0.259 mV
HA-CIP-SNEDDS	40 ± 0.457 nm	0.2 ± 0.003	−11.4 ± 0.465 mV

**Table 4:** Kinetic models to evaluate the mechanism of CIP drug release from polymeric SNEDDS.

Kinetic Models	Zero Order Ct = C0 + k0t		First Order logQ0 + Kit/2.3		Korsmeyer-Peppas Mi/M∞ = Kth + b		Higuchi f1 = Q = KH√t	
Formulation	R^2^	K^0^	R^2^	K_1_	R_2_	*N	R_2_	KH
CIP	0.5787 ± 2.57	32.90	0.9534 ± 1.57	0.877	0.9370 ± 4.85	0.502	0.9360 ± 3.57	53.70
CIP-SNEDDS	0.194 ± 2.67	7.095	0.9672 ± 2.07	0.286	0.9551 ± 3.59	0.360	0.8748 ± 2.47	26.157
HA SNEDDS	0.6227 ± 3.01	2.77	0.8358 ± 4.57	0.137	0.9765 ± 3.10	0.345	0.8768 ± 3.42	17.310
HA-CIP SNEDDS	0.5431 ± 3.17	2.29	0.8465 ± 2.59	0.060	0.9964 ± 2.97	0.437	0.9920 ± 2.57	13.45

**Table 5:** Results of in-vivo pharmacokinetic properties after oral delivery of CIP and HA-CIP SNEDDS to mice.

Parameter	Unit	CIP	HA-CIP
t_1/2_	H	4 ± 1.57	16 ± 3.57
T max	H	1 ± 3.37	3 ± 3.12
C max	μg/mL	54.50 ± 2.57	225.65 ± 3.89
AUC 0-t	μg/mL × h	210.75 ± 3.59	1562.83 ± 1.23
AUC 0-inf	μg/mL × h	600.84 ± 2.17	2124.50 ± 1.90
AUMC 0-inf	μg/mL × h^2^	900.65 ± 1.57	36,794.5 ± 2.68

## Data Availability

Not applicable.

## Supplementary Materials

The following are available online at https://www.mdpi.com/article/10.3390/nano11051086/s1, Figure S1: Final SNEDDS formulation [A], Solubility in various oils [B], surfactants and co-surfactants[C]. DLS of final SNEDDS formulation [D], Transmission electron micrographs of HA-CIP SNEDDS(a) and HA-SNEDDS(b) [E], SEM micrographs of HA-CIP-SNEDDS (a) HA-CIP polymer(b) [F]., Figure S2. DSC thermograms of CIP(a), HA(b) and HA-CIP(c).
