# Supplementary material for: A Hyaluronic Acid Functionalized Self-Nano-Emulsifying Drug Delivery System (SNEDDS) for Enhancement in Ciprofloxacin Targeted Delivery against Intracellular Infection
Source: Nanomaterials (Basel). 2021 Apr 22;11(5):1086. doi: 10.3390/nano11051086 (PMC8146397; doi:10.3390/nano11051086)
Supplement: Supplementary file 1 [file nanomaterials-11-01086-s001.zip › nanomaterials-1164343-supplementary.pdf]

Supporting materials

A Hyaluronic Acid Functionalized Self-Nano-Emulsifying Drug Delivery System (SNEDDS) for Enhancement in Ciprofloxacin Targeted Delivery against Intracellular Infection

Rabia Arshad [1], Tanveer A. Tabish [2], Maria Hassan Kiani [1], Ibrahim M Ibrahim [3], Gul Shahnaz [1,*], Abbas Rahdar [4,*], Misook Kang [5,*] and Sadanand Pandey [5,*]

[1] Department of Pharmacy, Quaid-i-Azam University, 45320 Islamabad, Pakistan; rabia.arshad@bs.qau.edu.pk (R.A.); marria.h.kyani@gmail.com (M.H.K.)
[2] UCL Cancer Institute, University College London, London WC1E6DD, UK; t.tabish@ucl.ac.uk
[3] Department of Pharmacology, College of Medicine, King Abdulaziz University, Jeddah, Saudi Arabia; imibrahim1@kau.edu.sa
[4] Department of Physics, Faculty of Science, University of Zabol, 538-98615 Zabol, Iran
[5] Department of Chemistry, College of Natural Science, Yeungnam University, 280 Daehak-Ro, Gyeongsan, Gyeongbuk 38541, Korea
* Correspondence: gshahnaz@qau.edu.pk (G.S.); a.rahdar@uoz.ac.ir (A.R.); mskang@ynu.ac.kr (M.K.); sadanand.au@gmail.com or spandey@ynu.ac.kr (S.P.)

1. Physicochemical Characterization

1.1.Percentage transmittance

Nano emulsions (1 ml) of CIP-SNEDDS and HA-CIP-SNEDDS was 100 times diluted in the distilled water. The transmittance was analyzed at 270 nm by using UV–visible spectrophotometer [1].

1.2. Dispersibility test

SNEDDS formulation was dissolved in distilled water (100 ml) and get stirred with stirring speed of 50 rpm at actual laboratory temperature on the magnetic stirrer hot plate. Time duration required to achieve the conversion of milky emulsion into the clear dispersion was recorded [2].

1.3. Saturation solubility

Polymeric SNEDDS were accessed for saturation solubility *via* using shake flask method. Optimized polymeric SNEDDS containing excessive amount of CIP was vortexed for 10 minutes and then homogenized for 48 h by 50 rpm stirring speed at 37°C in shaking water bath [3].

1.4. Robustness to dilution

The optimized polymeric SNEDDS formulation was diluted in the ratios if 1:50, 1:100, 1:1000 times. The formed nano emulsions were placed for 24 h and accessed for transmittance, coalescence, clarity and phase separation [4].

1.5. Cloud point determination

Temperature on which the clear nano emulsion turns turbid is known as cloud point temperature. The HA-CIP-SNEDDS formulation dilutions in ratio 1:100 was heated on the hot plate with a gradual increase (2°/min) (from 25 to 60 °C). The point where clear nano emulsion suddenly turned into cloudy emulsion was recorded as the cloud point temperature [5].

2. Results

However, it was obvious from results that all optimized SNEDDS formulations showed the highest transmittance. Fine and stable nano emulsions with no signs of phase separation were obtained even after dilutions with deionized water at the ratio of 1:100 and 1:1000.

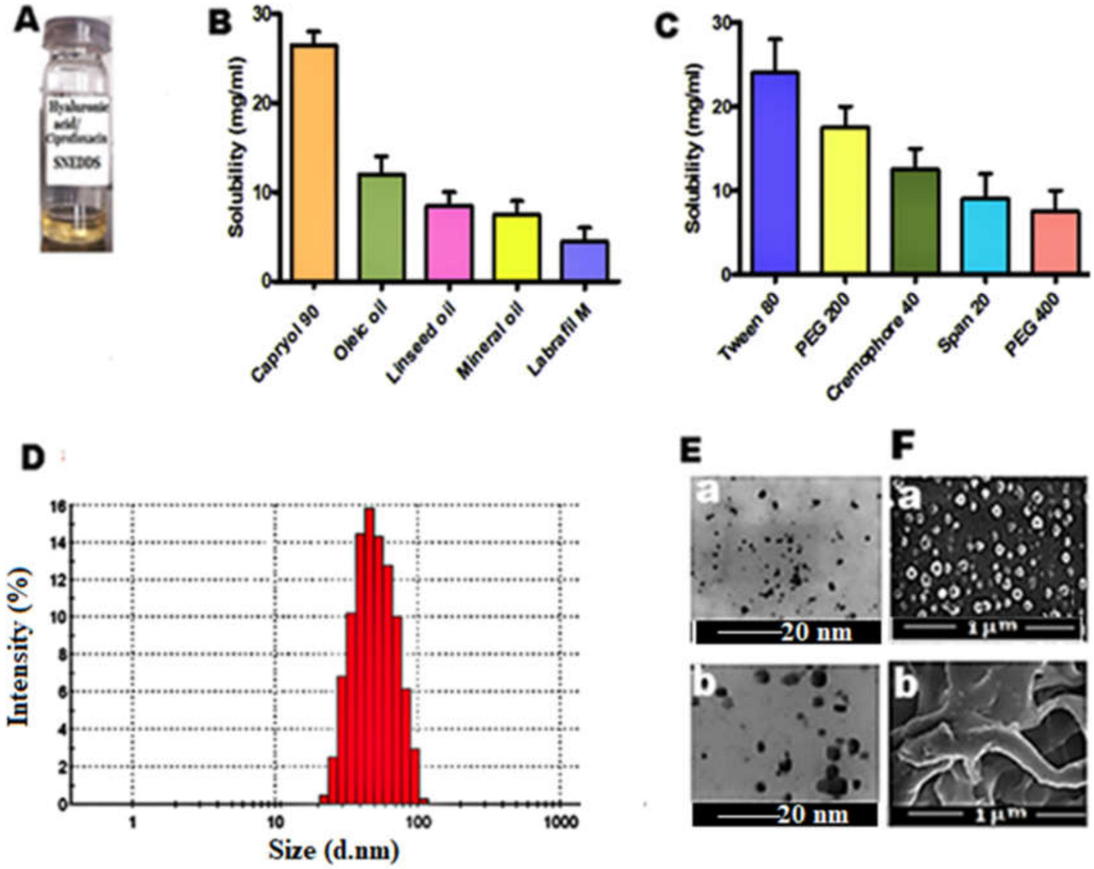

Figure S1. Final SNEDDS formulation [**A**], Solubility in various oils**[B]**, surfactants and co-surfactants**[C]**. DLS of final SNEDDS formulation [**D**], Transmission electron micrographs of HA-CIP SNEDDS(**a**) and HA-SNEDDS(**b**) [**E**], SEM micrographs of HA-CIP-SNEDDS (**a**) HA-CIP polymer(**b**) [**F**].

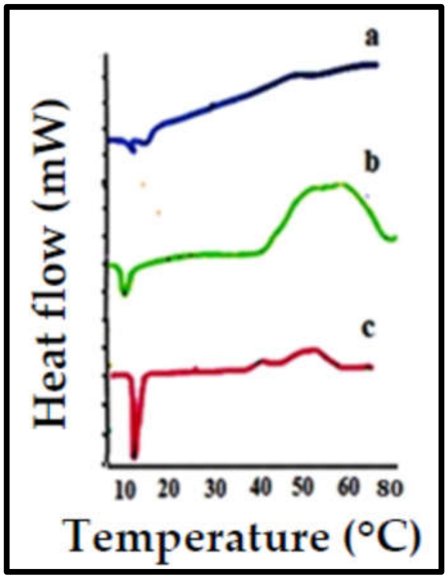

Figure S2. DSC thermograms of CIP(**a**), HA(**b**) and HA-CIP(**c**).

References

1. Abouhussein, D.M., D. Bahaa El Din Mahmoud, and E. Mohammad F, Design of a liquid nano-sized drug delivery system with enhanced solubility of rivaroxaban for venous thromboembolism management in paediatric patients and emergency cases. *J. Liposome Res.*, 2019. **29**(4): p. 399-412.
2. Shakeel, F., et al., Thermodynamic modeling for solubility prediction of indomethacin in self-nanoemulsifying drug delivery system (SNEDDS) and its individual components. *Drug Dev Ind Pharm*, 2014. **40**(9): p. 1240-1245.
3. Shakeel, F., et al., Thermodynamics and solubility prediction of talinolol in self-nanoemulsifying drug delivery system (SNEDDS) and its oil phase components using mathematical modeling. *J Drug Deliv Sci Technol*, 2014. **24**(5): p. 533-538.
4. Syukri, Y., et al., Novel Self-Nano Emulsifying Drug Delivery System (SNEDDS) of andrographolide isolated from Andrographis paniculata Nees: characterization, in-vitro and in-vivo assessment. *J Drug Deliv Sci Technol*, 2018. **47**: p. 514-520.
5. Kim, J.S., et al., Comparative study between high-pressure homogenisation and Shirasu porous glass membrane technique in sildenafil base-loaded solid SNEDDS: Effects on physicochemical properties and in vivo characteristics. *Int. J. Pharm.*, 2020: p. 120039.
